# Extraction and Analysis of Phenolic Compounds in Rice: A Review

**DOI:** 10.3390/molecules23112890

**Published:** 2018-11-06

**Authors:** Marco Ciulu, Maria de la Luz Cádiz-Gurrea, Antonio Segura-Carretero

**Affiliations:** 1 Department of Animal Sciences, Division of Quality of Animal Products, University of Göttingen, Albrecht-Thaer-Weg 3, 37075 Göttingen, Germany; 2 Department of Analytical Chemistry, University of Granada, c/Fuentenueva s/n, 18071 Granada, Spain; mluzcadiz@ugr.es (M.d.l.L.C.-G.); ansegura@ugr.es (A.S.-C.); 3 Research and Development of Functional Food Centre (CIDAF), PTS Granada, Avda. Del Conocimiento 37, Edificio BioRegion, 18016 Granada, Spain

**Keywords:** rice, phenolic compounds, phenolic acids, flavonoids, anthocyanins, proanthocyanidins, antioxidant activity, extraction, HPLC methods

## Abstract

Rice represents the main source of calorie intake in many world countries and about 60% of the world population include rice in their staple diet. Whole grain rice, also called brown rice, represent the unpolished version of the more common white rice including bran, germ, and endosperm. Many health-promoting properties have been associated to the consumption of whole grain rice and, for this reason, great attention has been paid by the scientific community towards the identification and the quantification of bioactive compounds in this food item. In this contribution, the last five years progresses in the quali-quantitative determination of phenolic compounds in rice have been highlighted. Special attention has been devoted to the most recent strategies for the extraction of the target compounds from rice along with the analytical approaches adopted for the separation, identification and quantification of phenolic acids, flavonoids, anthocyanins, and proanthocyanidins. More specifically, the main features of the “traditional” extraction methods (i.e., maceration, ultrasound-assisted extraction) have been described, as well as the more innovative protocols involving advanced extraction techniques, such as MAE (microwave-assisted extraction). The predominant role of HPLC in the definition of the phenolic profile has been examined also presenting the most recent results obtained by using mass spectrometry-based detection systems. In addition, the most common procedures aimed to the quantification of the total amount of the cited classes of phenolic compounds have been described together with the spectrophotometric protocols aimed to the evaluation of the antioxidant properties of rice phenolic extracts (i.e., FRAP, DPPH, ABTS and ORAC).

## 1. Introduction

Cereals play a meaningful role in the human diet not only for the wide variety of nutrients provided but also for their significative caloric contribution. Among cereals, rice is nowadays one of the most important from both a nutritional and an economic point of view since about 60% of the world’s population include rice in their basic diet. More specifically, rice is the most cultivated crop in the Asian-Pacific region and it constitutes the staple food in several developing countries such as India, Bangladesh, Vietnam, etc. [1].

Brown rice, also called whole grain rice, is the result of the removal of the inedible outer hull and, unlike the common white rice, it includes the germ, bran, and endosperm. Depending on the various pigmentations of the outer layer, whole grain rice can be classified as black, purple, red, etc. Several health-promoting and nutraceutical properties have been associated to the consumption of whole grain rice [2]. For instance, in the study conducted by Hallfrisch and co-workers (2003) a decrease of the cardiovascular risk was observed because of enhancement of the intake of whole grain foods in the daily diet [3]. Moreover, the consumption of pre-germinated brown rice as staple diet proved to produce a positive effect in the countering of depression, hostility and fatigue in a group of breast-feeding mothers [4]. In addition, the antidiabetic properties of germinated brown rice have been reviewed by Imam et al. (2012) [5]*.* Many of the documented health benefits of brown rice have been associated to the occurrence of polyphenols in its chemical composition. It appears clear that the presence of phenolics in rice represents an added value for this food item whose inclusion in the daily diet is relevant not only for its nutritional content, but also for the related health benefits. Most of phenolic compounds traceable in rice are present in insoluble bound forms and only colonic digestion is able to facilitate the release of these compounds from cell wall materials [6]. Instead, the other fractions include the free and soluble conjugate forms.

**Figure 1:**
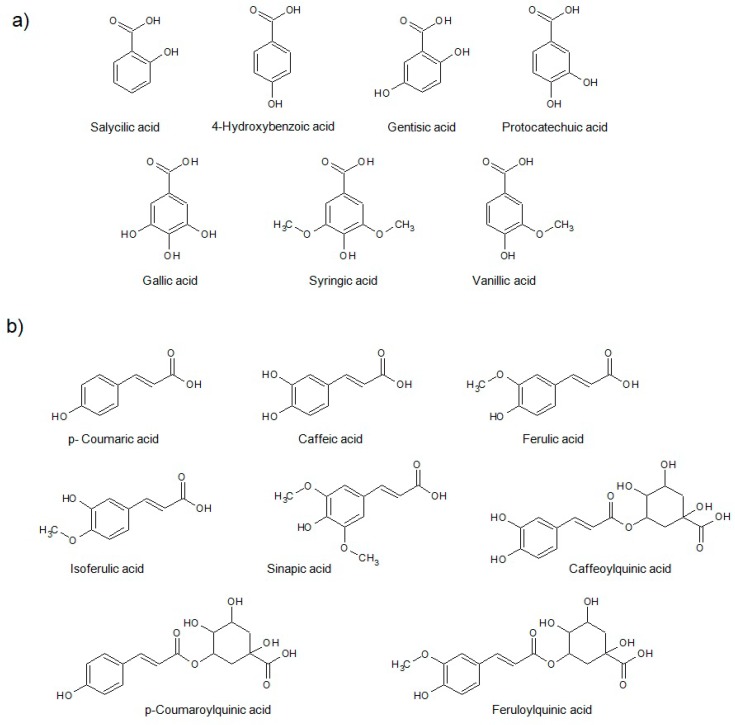
Hydroxybenzoic (**a**) and hydroxycinnamic acids (**b**) commonly found in rice.

**Figure 2:**
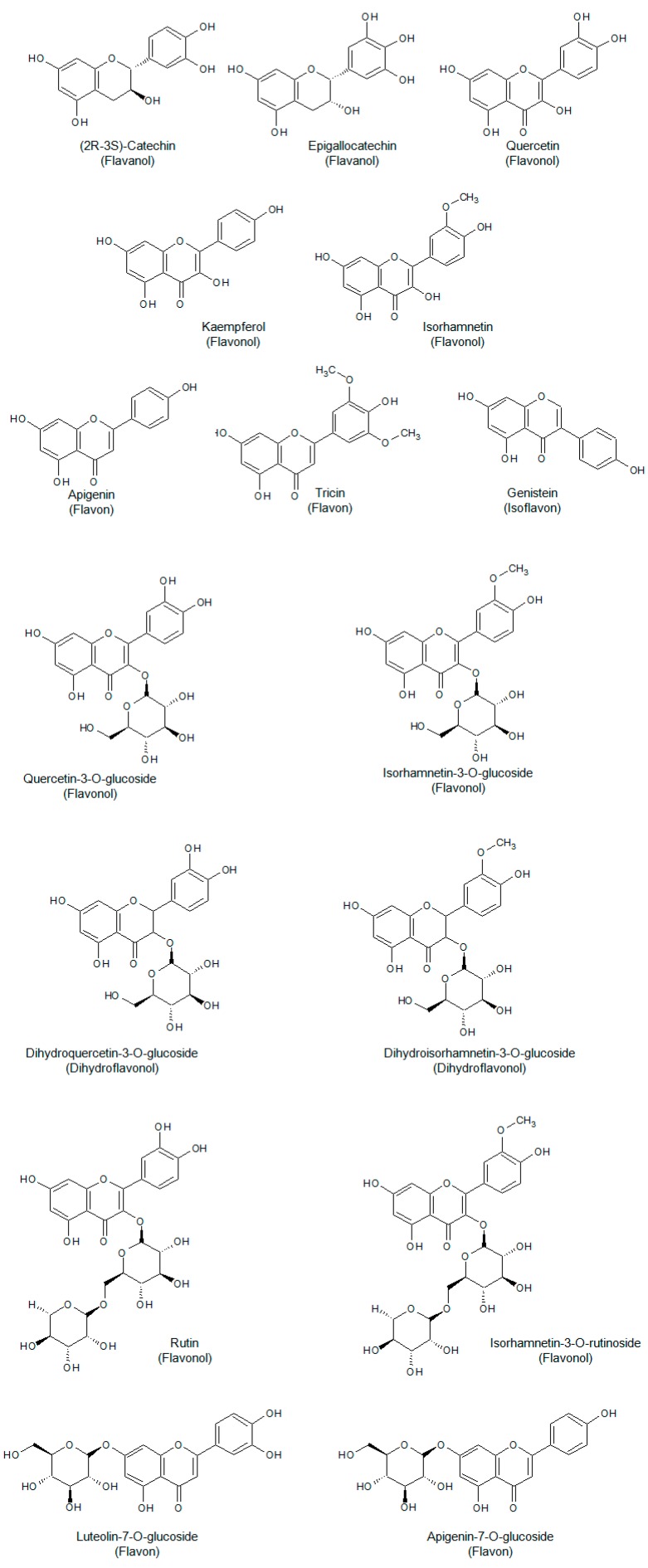
Some flavonoids commonly found in rice.

Phenolic acids constitute an important class of the phenolic fraction of brown rice, including both hydroxybenzoic and hydroxycinnamic acids. Figure 1 shows the structure of some common phenolic acids detected in rice. Additionally, flavonoids, whose structure is characterized by the presence of an *x*-phenyl-1,4-benzopyrone backbone (where *x* = 2, 3), are a noteworthy part of the diverse phenolic fraction of rice. As illustrated in Figure 2, the most common flavonoids of rice belong to a wide variety of subfamilies such as flavonols, flavones, flavanols, flavanons, isoflavones, etc.

**Figure 3:**
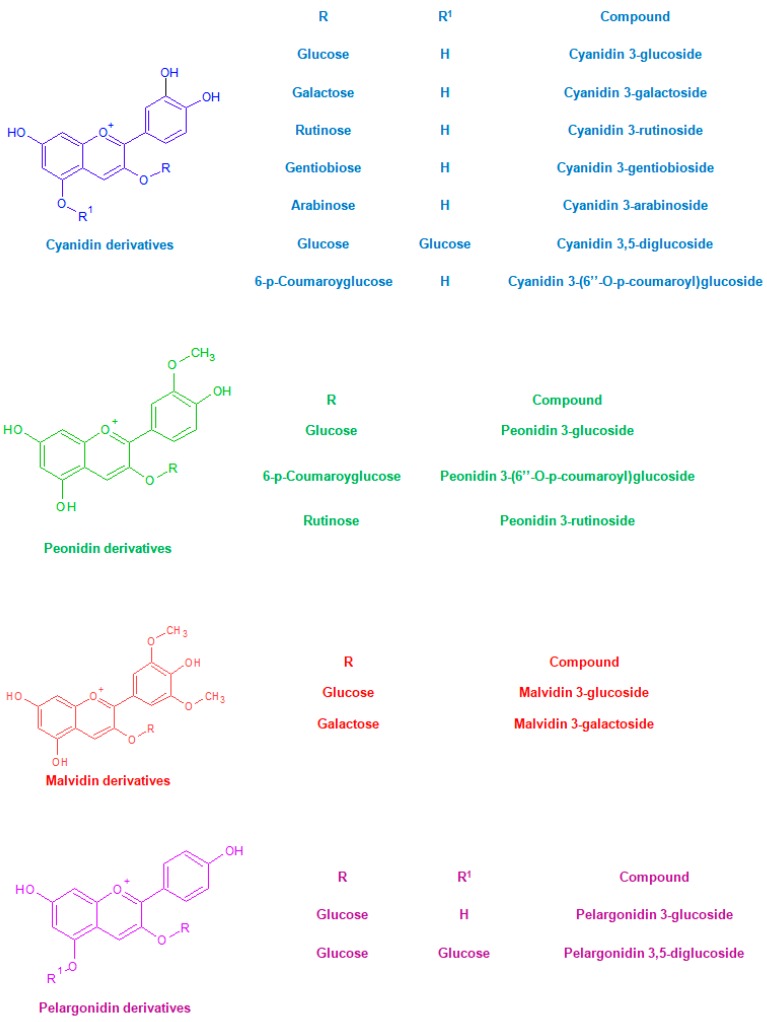
Some common anthocyanins detected in rice [2].

Phytochemical studies on whole grain rice brought to light many interesting aspects related to the presence of anthocyanins. This peculiar subgroup of polyphenols is noteworthy not only for being water-soluble pigments who contribute to the colour of fruit and vegetables, but also for their several well-documented health-promoting properties [7]. In vitro experiments performed on human cells proved the ability of anthocyanin extracts from black rice to inhibit the motility of cancer cells of various types [8]. In addition, this type of extracts seems to reduce platelet hyperactivity, hypertriglyceridemia and body weight gain in male dyslipidemic rats [9]. As shown by Figure 3, derivatives of cyanidin, peonidin, malvidin and pelargonidin have been traced in rice [2]. Finally, the phenolic profile of red rice is characterized by the presence of proanthocyanidins. These oligomeric polyphenols, which include in their structure mainly (+)-catechin and (−)-epicatechin monomeric units [2], proved to possess a considerable antioxidant activity [10].

On the basis of the above, it is evident that the analytical determination of the quali-quantitative profile of polyphenols is a necessary prerequisite not only to define the nutritional qualities of whole grain rice, but mostly to investigate on the health benefits associated to the consumption of this food item. The broad variety of structures of the target analytes and the usually low concentration levels (sometimes fractions of mg/100 g dry weight, [2]) imply the adoption of precise and accurate multistep analytical strategies able to correctly isolate, identify and quantify these bioactive compounds in a complex food matrix. In addition, in order to assess the reliability of the quantitative data a complete validation protocol is needed for the proposed procedures.

Scientific literature offers several examples related to the quali-quantitative determination of polyphenols in whole grain rice along with the evaluation of the phenolic compounds-related properties (i.e., antioxidant activity). At best of our knowledge, no recently published review provides a comprehensive view on the state of the art regarding the analytical methods adopted for the definition of the phenolic profile of brown rice and the assessment of the bioactivity of the extracts. Hence, the primary goal of this contribution is to highlight the recent advances in this field focusing the attention on the studies carried out during the last five years. Special attention will be devoted to the extraction procedures aimed to the isolation of the target analytes, the chromatographic conditions adopted to properly outline the phenolic profile, the spectrophotometric protocols devoted to the quantification of the total content of specific classes of polyphenols and also to the determination of the antioxidant properties.

## 2. Extraction and Clean-Up

Extraction is a key step in the determination of phenolic compounds in rice. The main challenge is represented by the necessity to isolate compounds belonging to different classes (i.e., phenolic acids, flavonoids, proanthocyanidins etc.) and to remove possible interferences. Moreover, the choice of the most suitable extraction procedure strongly depends not only on the type of analytical information that has to be acquired (i.e., quali-quantitative profile, total content of polyphenols, total content of flavonoids, antioxidant activity, etc.) but also on the nature of the phenolic fractions that will be quantified (i.e., free and/or bound phenolics) [11,12].

Generally, a sample pre-treatment step is required before the extraction. Rice is usually subjected to a drying process aimed to stabilize the samples preventing the microbial spoilage and the hydrolytic rancidity [13]. In most cases, this operation is performed by means of common air-dryers [10,14,15,16,17,18] but the decrease of moisture can be also achieved by sun-drying [19], microwave heating [13], superheated steam fluidized bed drying [20], or far-infrared radiations (FIRs) exposure [21]. More specifically, the FIR treatment has proved to be particularly advantageous of the extraction of polyphenols from rice by-products [21]. In addition, sterilization of rice samples can be achieved by washing with a 1% (*v*/*v*) sodium hypochlorite solution [22].

Rice samples can be dehulled by the use of laboratory milling machines [23], degermed, and/or polished, depending on the part of the whole grain which has to be studied (brown rice, white rice, bran etc.). Finally, the sample is generally ground to a fine powder in order to obtain a homogeneous material, which can be sometimes freeze-dried and stored in darkness at 4 °C [16].

In some cases, prior to extraction lipids are removed from the sample by means of hexane [13,24]. The breaking of the bonds between the phenolic compounds and the insoluble polymers of the cell walls can be enhanced by the use of specific enzymes. For example, in the study by Wanyo et al. (2014), it was demonstrated that enzymatic hydrolysis with cellulase of the rice husk can significantly increase the total phenolic content of the extracts [21].

As for the actual extraction phase, maceration is the most adopted procedure for the isolation of rice phenolic compounds. In the last years, a variety of polar solvent mixtures has been employed for this aim depending on the nature of the target analytes. For example, according to the study performed by Alves et al. [25], the mixture acetone/water (70/30, *v*/*v*) provides the best results for the extraction of free phenolics from black, red, and wild samples and increases the recovery of anthocyanins and proanthocyanidins from red rice. On the other hand, the mixture acetone/water/acetic acid (70/29.5/0.5, *v*/*v*/*v*) proved to be the best option for the isolation of anthocyanins from pigmented rice indicating, in this specific case, the important role played by the acidification of the solvent system. In addition, the number of extraction steps has a strong influence on the recovery of the analytes. In fact, according to the same study, at least three extraction steps should be performed to quantitatively extract free phenolics.

Extraction of phenolic compounds from rice can be sometimes improved by the use of ultrasounds [14,26,27,28]. In fact, it is well known that the application of ultrasounds promotes the diffusion of the phenolic compounds from the vegetal cells to the solvent medium [29,30,31].

**Figure 4:**
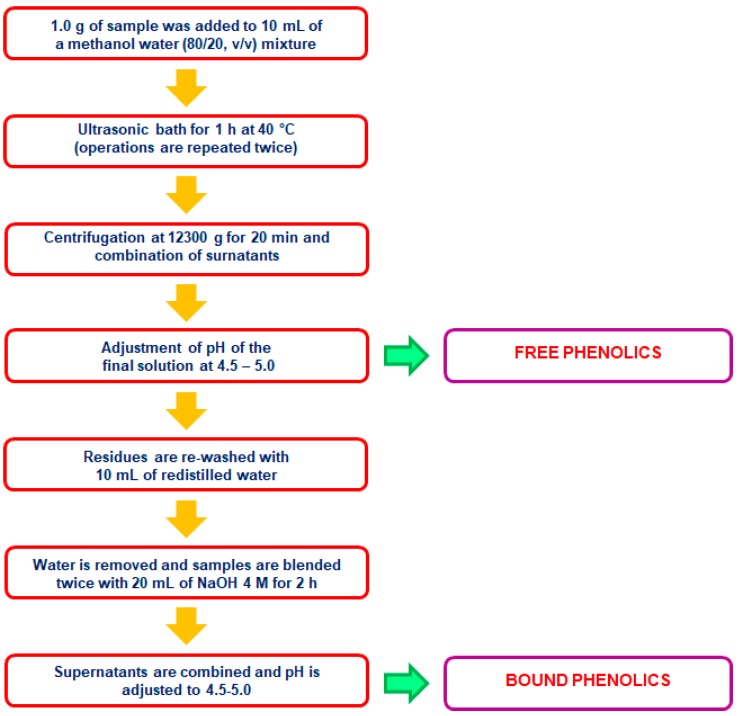
Extraction of free and bound phenolics as described by Sumczynski et al. (2017) [14].

As with other food products [32,33], an additional step is usually required when the study involves the extraction of the free and bound phenolic fractions (Figure 4). Generally, for the extraction of the bound (ester and/or ether-linked) phenolics, a hydrolysis step with NaOH is perfomed after the isolation of the free fraction. Recovery of bound phenolic fraction can be optimized by the use of α-amylase thanks to the reduced viscosity of the residue during the alkaline hydrolysis [25].

**Figure 5:**
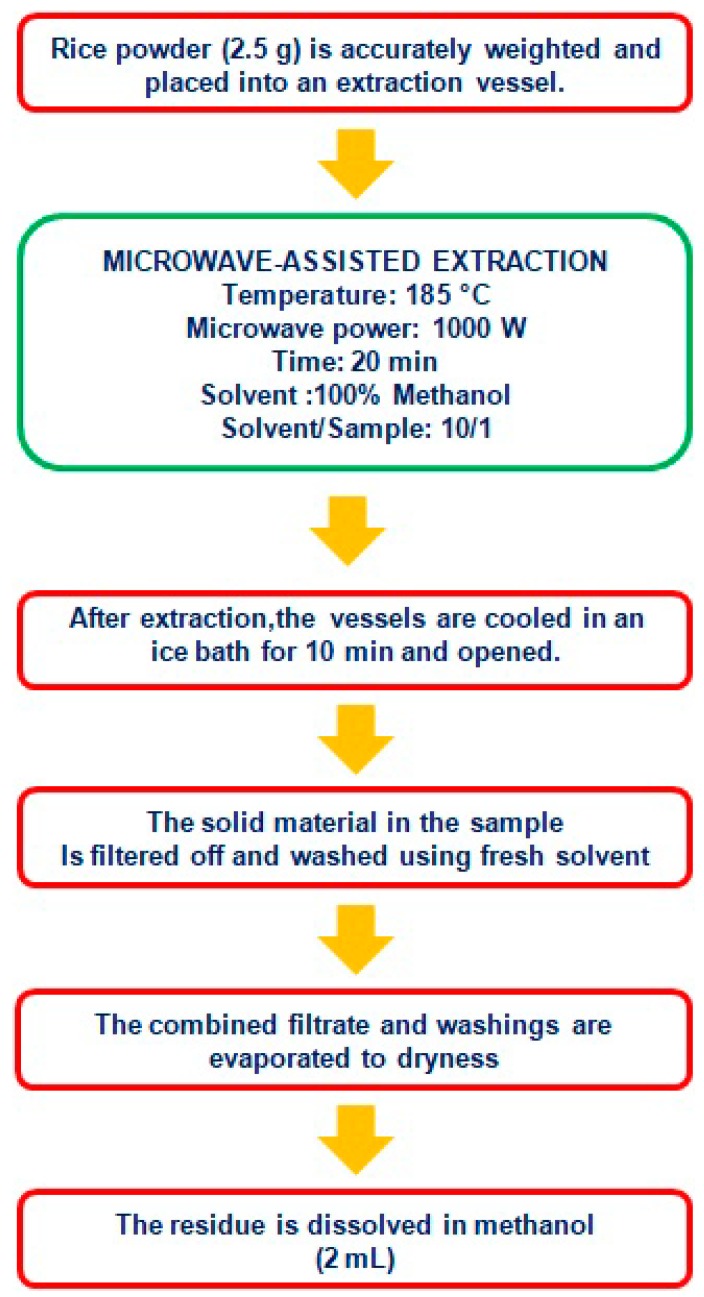
Microwave assisted extraction (MAE) of phenolic compounds from rice as described by Setyaningsih et al. (2015) [34].

In the study by Setyaningsih et al. (2015) [34] the first example of application of the microwave radiation for the extraction of phenolic compounds from rice was reported. In this case, the extraction of the analytes is promoted by the break of the weak hydrogen bonds due to the dipole rotation of the molecules. One of the main advantages of the microwave-assisted extraction (MAE) is represented by the possibility to obtain phenolic compound-rich extracts in a short time. In the cited work, for instance, the target compounds were extracted from the rice grains in only 20 min. This represents a big improvement considering that the more conventional maceration generally requires hours to achieve a satisfactory extraction. A scheme of the analytical procedure optimized and validated by the authors is reported in Figure 5. Lastly, as reported by SantiStefanello et al. (2018) the MAE of polyphenols from rice bran performed with alkaline solution showed the best results in terms of total phenolic content when compared to the traditional solid-liquid extraction [35].

## 3. Analysis of the Quali-Quantitative Profile of Phenolic Compounds in Rice

The definition of the quali-quantitative profile of polyphenols in rice involves the separation, the identification and the quantification of the analytes extracted in the previous steps. In this context, HPLC represents the most adopted analytical technique. Selected features of recent chromatographic methods for the analysis of phenolic compounds in rice have been summarized in Table 1.

As for the extraction phase, the main obstacle in the quali-quantitative determination of polyphenols in rice is represented by the need to assess chromatographic conditions which can be optimal for analytes belonging to different classes. Although it is difficult to find any kind of uniformity in the broad variety of analytical methods recently proposed, it is safe to say that when both phenolic acids and flavonoids have to be analysed, a single chromatographic run is generally adopted for the separation [14,15,16,35,36]. In this case, C18 columns are usually preferred but analysis can be also performed by means of ODS [18,37] and phenyl columns [38]. As for the mobile phase, this is generally composed of the gradient of two solvents: (A) an aqueous solution of acetic acid [14,15,17,21,39] or alternatively, trifluoroacetic acid [16,40] or formic acid [10,37], and (B) acetonitrile or methanol, sometimes acidified with acetic acid or trifluoroacetic acid. Occasionally, more elaborated elution programs involving the use of three different solvents are used for the simultaneous determination of phenolic acids and flavonoids [27,35].

On the other hand, proanthocyanidins and anthocyanins are usually analysed with alternative chromatographic methods. As in the case of phenolic acids and flavonoids, separation of the analytes is generally achieved by the use of reverse-phase columns. Gradient elution is performed with a binary solvent system where formic acid is usually dissolved in water for the solvent A while methanol (or acetonitrile), occasionally acidified, is adopted for the second eluent (solvent B). In the study by Phetpornpaisan et al. (2014) [41] the simultaneous analysis of phenolic acids and anthocyanins (limited to cyanidin 3–glucoside and its corresponding aglycone) is made possible by the use of a CN stationary phase and a gradient mobile phase comprised of phosphoric acid, water, and acetonitrile. Unfortunately, in the cited study the elution program chosen for the separation of the analytes is not reported.

As concerns proanthocyanidins, the oligomeric nature of these analytes sometimes implies the assessment of ad hoc procedures. Whilst in some cases monomers and dimers [27], or alternatively dimers and trimers [10], are separated by means of a C18 column, an example of normal phase chromatography was proposed by Min et al. (2014) [36] for the quantification of proanthocyanidins oligomers (from monomers to decamers).

UV absorption still remains the most exploited property for the detection of rice phenolic compounds. In most cases, diode array detectors are employed thanks to their capacity to acquire the complete UV–VIS spectra of the analytes and the consequent possibility to choose the most convenient wavelength for the revelation. In fact, as already explained, the main challenge is represented by the need to assess the best detection parameters for compounds usually characterized by different chemical structures and belonging to various classes. Nevertheless, this type of detector is strongly limited when it comes to the identification and the correct attribution especially when commercial standards are not available. For this reason, in recent years many studies regarding the phenolic fraction of rice involved the use of mass spectrometry-based techniques. The electro-spray ionisation (ESI) source is normally installed in this kind of equipment and it is generally set on the negative mode for the detection of phenolic acids and flavonoids and on the positive mode for anthocyanins. Nonetheless, also atmospheric pressure chemical ionization (APCI) proved to be successful for the MS-based characterization of phenolic acids and proanthocyanidins [10].

The exploitation of mass spectrometry and tandem mass spectrometry as detection systems combined with HPLC allowed making progress in the study of the phenolic fraction of rice. More specifically, some phenolic compounds have been traced for the first time in this food matrix thanks to the information revealed by the fragmentation pattern. For example, in the study proposed by Huang and Lai (2016) [42], protocatechualdehyde was identified for the first time in the bound phenolic fraction of red rice by means of LC-MS/MS. In addition, as showed by the work conducted by Bordiga et al. (2014) [27], the possibility to perform MS^3^ targeted experiments results to be extremely useful to elucidate the structure of peculiar molecular species such as flavanol-anthocyanins adducts (catechin/epicatechin monomer linked to cyanidin-3-glucoside or peonidin-3-glucoside). Compounds whose discrimination is made difficult by strong similarities in their structures can also be characterized thanks to the potential offered by MS detectors. In the study proposed by Shao et al. (2014) [19], for instance, *cis*-isomers of sinapic and *p*-coumaric acids were identified and distinguished from the respective *trans* forms in the bound phenolic fraction of rice.

A more traditional approach for the identification of phenolic compounds in rice is proposed in the study by Wang and co-workers (2015) [43]. In this case, the ethyl acetate rice extract is separated into sub-fractions by using a normal-phase silica gel column. The sub-fractions are subsequently characterized by NMR and ESI-MS spectroscopy. The experimental work led to the identification of 10 compounds including some ferulic acid derivatives, methyl caffeate, vanillic aldehyde, and *p*-hydroxy methyl benzoate glucoside, which was isolated for the first time in rice.

In addition to liquid chromatography, gas-chromatography (GC) can be also sometimes employed to investigate the quali-quantitative profile of phenolic compounds of rice. Although HPLC methods are generally the most indicated for the identification and the quantification of phenolics, some interesting results have also been obtained by GC. Kim and co-workers (2013) [44], for example, performed the classification of different rice cultivars by principal component analysis (PCA) using GC-TOF-MS data. In this specific case, it is important to highlight the fact the authors adopt a type of approach which is quite innovative for the definition of the quali-quantitative profile of phenolics in rice. In fact, while most authors decide to follow a target approach aimed to assess extraction and separation conditions optimized for specific classes of compounds, in the cited study all the polar metabolites detectable with the selected technique (i.e., GC-TOF-MS) are considered. In the study by Yodpitak et al. (2013) [45] GC-MS was exploited to demonstrate how the application of a pre-germination process can positively influence the concentration of antioxidant compounds in brown rice.

It is crucial to remember that when the study involves not only the identification but also the quantification of the phenolic compounds in rice (or in other matrices) validation is essential to express the reliability of quantitative data. According to the guidelines provided by ISO/IEC 17025 [46], a method should be validated whenever it is: (a) a non-standard method; (b) a laboratory-designed (or developed method); (c) a standard method used outside its intended scope; (d) an amplification and/or modification of standard method. Also some international organizations like FAO [47] or EC [48] require to evaluate the analytical performances of the procedures applied to the food sector. More specifically, the proposed methods should be evaluated in terms of sensitivity (providing LoDs and LoQs), working range, precision and trueness. Unfortunately, most of the methods indicated in Table 1 are completely invalidated so that some doubts on the reliability of the data should be raised.

## 4. Total Content of Polyphenols, Flavonoids, Proanthocyanidins, and Anthocyanins

In addition to the determination of the quali-quantitative profile of phenolics, it is also possible to achieve the quantification of the total content of specific classes of compounds. In this context, it is absolutely noteworthy to mention the Folin-Ciocalteu’s procedure for the determination of the total phenolic content. This represents the most adopted protocol for the global quantification of phenolics, not only in rice, but also in almost all the food and vegetal matrices. The colorimetric method is based on the formation of molybdenum- and tungsten-based blue-coloured complexes that can be spectrophotometrically revealed. The procedure, as described for example by Walter et al. (2013) [50], involves the dilution of an aliquot of the phenolic extract in distilled water and the addition of the Folin-Ciocalteu’s reagent. Spectrophotometry can be also employed to quantify the total content of flavonoids in rice. The most widely adopted procedure exploits the complexation of the analytes with aluminium chloride (AlCl_3_) and the consequent hyperchromic effect provided by the formed complex [42] As for the total condensed tannins (proanthocyanidins), this parameter is generally determined by the vanillin assay. In this case, an aromatic aldehyde (vanillin) reacts with the meta-substituted ring of flavanols to produce a red adduct which can be spectrophotometrically detected. The method, as described by Gunaratne et al. (2013) [10] involves the mixing of a small volume of methanolic extract with a solution of sulphuric acid/methanol and a 1% methanolic solution of vanillin. The mixture is heated at 30 °C for 15 min with a water bath and then the absorbance is read at 500 nm against the blank. Catechin is adopted as external standard and results are expressed as mg of catechin per g of dry matter. Since anthocyanins can interfere with the determination, a control mixture should be prepared by adding 100% methanol in place of the vanillin reagent in order to correct the absorbance.

Finally, for the quantification of total anthocyanins (TAC) authors sometimes choose to measure the absorbance of the extracts at a specific wavelength and calculate the TAC using the molar absorptivity of an anthocyanin commonly found in rice such as cyanidin-3-glucoside [23,36]. In other cases, a pH differential protocol is preferred. The method is based on the structural change of the anthocyanin chromophore between pH 1 and 4.5. In the study by Ti et al. (2015) [17] the extracts are mixed with two different buffers at pH 1 and 4.5 respectively, and the absorbance of each solution is measured at 515 nm and 700 nm. The TAC is calculated considering the difference of the absorbance at the selected wavelengths and using, also in this case, the molar absorptivity of cyanidin-3-glucoside.

Even though all the described spectrophotometric procedures are recognized as the most indicated to quantify the total content of the selected classes of polyphenols in rice, it is extremely important to remember that they are strongly aspecific. For example, the determination of the total phenolic content by means of the FC protocol could be significantly affected by the presence of other reducing substances such as proteins, amino acids etc. Moreover, similarly to what previously described for the quali-quantitative profile, also in this case the reliability of data can be questioned because of the complete absence of a validation protocol. This fact, along with the dissimilarities in the extraction and calibration modalities, make extremely challenging the potential comparison among data coming from different studies. For instance, when quantification of total flavonoids is achieved using different compounds for the calibration (i.e., catechin or quercetin), data are obviously incomparable. Anyway, results obtained by means of the cited procedures could be useful when comparison is performed in the same context and among data related to the same type of extracts and deriving from homogeneous calibration curves.

## 5. Antioxidant Activity Assays

Reactive free radicals have been postulated to contribute to the causes of chronic inflammatory proliferative diseases, especially arteriosclerosis and cancer, through oxidative damage of essential enzymes, cells, and tissues [51]. Unique phytochemicals present in grains, which complement those in fruits and vegetables when consumed together, have antioxidant properties associated with the health benefits of grains and grain products. As already explained, these phytochemicals include various classes of phenolic compounds, which may exist in free, soluble conjugate and/or insoluble bound forms. Despite the importance of this subject, there is a limited amount of literature dealing with the relation between the phenolic fractions and the antioxidant activity of rice, as well as with the contribution of individual phenolics to the antioxidant properties.

Some in vitro and in vivo studies have demonstrated the antioxidant effect of rice by different methods. These can be classified into two main categories: single electron transfer (SET) and hydrogen atom transfer (HAT) assays. As regards the first ones, the evaluation of 2,2’-azinobis-(3-ethylbenzo-thiazoline-6-sulfonic acid) cation (ABTS^+^) scavenging activity has been widely applied to rice extracts. This is a decolorization assay applicable to both lipophilic and hydrophilic antioxidants. For instance, in the study by Re et al. (1999), the pre-formed radical monocation of 2,2′-azinobis-(3-ethylbenzothiazoline-6-sulfonic acid), ABTS^•+^ is generated by oxidation of ABTS with potassium persulfate and is reduced in presence of antioxidants. The influence of both concentration of antioxidants and duration of reaction are taken into account when determining the antioxidant activity [52]. This methodology was also followed by Ti et al. (2014) in order to study the ABTS radical scavenging activity of different varieties of conventional and hybrid rice grown in southern China. In this scenario, the authors mixed the aqueous solutions of ABTS and potassium persulfate and left the mixture in the dark at room temperature for 12–16 h before use. An aliquot of rice extract was then added to a diluted methanolic solution of ABTS radical. The absorbance was measured at 734 nm and the ABTS antioxidant activity was expressed as µM Trolox equivalents per g DW. The results of this research showed that the antioxidant activity of bran from different rice varieties was much higher than that in polished rice [39]. Surprisingly, among the various proposed studies regarding the ABTS evaluation, the reaction time appears to be extremely variable. For example, in the work by Chan et al. (2013) a reaction time of 10 min is adopted for the estimation of the ABTS scavenging activity of defatted bran [13] whereas in the study by Sompong and co-workers (2011) the absorbance of the final mixture is read after only 1 min [53].

Another SET protocol that is widely applied to rice extracts is the 2,2-diphenyl-1-picrylhydrazyl (DPPH) free radical scavenging method. Shao and Bao (2015) determined the antioxidant capacity of three rice accessions, grown at the farm of Zhejiang University, by this analytical procedure [2]: appropriately diluted crude sample extracts were added to 3 mL of a methanolic DPPH solution. After incubating for 30 min in the dark, the absorbance was measured by a spectrophotometer at 517 nm. Also in this case, some differences can be found in the DPPH procedures described by the various authors especially as concerns reaction time. In fact, while most of authors decide to keep the final mixture in the dark for 20 min, some studies don’t provide details for this aspects [24,42,54]. Concerning the absorbance, this is generally measured at 515 or 517 nm. Additionally, 540 nm has also been adopted as a detection wavelength [13].

While ABTS and DPPH methods are both decolorization-based assays, the quantification of ferric reducing antioxidant power (FRAP), is based on the absorbance increase at a prespecified wavelength which takes place when the antioxidant compounds react with a chromogenic reagent [55]. The FRAP assay is generally considered as a robust, sensitive, simple and fast method that facilitates experimental and clinical studies investigating the relationship among antioxidant status, dietary habits, and risk of disease. The procedure, as described by most authors, involves the adding of an aliquot of rice extract to the FRAP solution which is generally prepared as described by Benzie and Strain (1996) [56]. For instance, the FRAP antioxidant activity of Brazilian rice cultivar samples was evaluated mixing an aqueous solution of 2,4,6-tripyridyl-s-triazine (TPTZ) and a solution of ferric chloride in sodium acetate buffer (pH 3.6), and then adding the rice extract [57].

As regards HAT-based methods, the oxygen radical absorbance capacity (ORAC) assay has been reported in some studies regarding the evaluation of nutraceutical properties of rice extracts, although this methodology is not very common as the others previously described and only few authors include it [36,58,59]. The ORAC assay is based on the peroxyl radical-induced oxidation initiated by thermal decomposition of azocompounds such as [2,2’-azobis(2-amidinopropane) dihydrochloride (AAPH)]. The reaction between this radical and a fluorescent probe (i.e., fluorescein) produces a decrease in the fluorescence intensity. When antioxidant compounds are present in the reaction mixture, a more stable fluorescence signal is observed during time thanks to the inhibition of the peroxyl radical. For example, in the procedure described by Càceres et al. (2014) for methanolic rice extracts, the reaction mixture contained fluorescein, 2,2-azo- bis(2-methylpropionamidine) dihydrochloride (AAPH) and the diluted sample. The reaction was carried out at 37 °C in phosphate buffer (pH 7.4) for 150 min. The fluorescence intensity was measured every minute (λ_exc_ = 485 nm; λ_emi_ = 520 nm) and the areas under the fluorescence decay curve (AUC) were recorded after subtracting the blank. The antioxidant activity values provided by the application of this assay allowed to optimize germination time and temperature for brown rice cultivars [60].

## 6. Conclusions

Whole grain rice is a high nutritional value food and it is included in the basic diet of the population of several countries. The presence of phenolic compounds in its chemical composition raised a great interest in this staple food also thanks to the health-promoting properties documented in various studies. Taking this into account, many groups have intensified their efforts to develop analytical methods aimed to identify and quantify polyphenols in rice, but also to estimate some of the nutraceutical properties deriving from the occurrence of these interesting compounds.

As regards the extraction of the target analytes, the traditional maceration with polar mixtures remains the most adopted technique although also some studies involving the use of ultrasounds and microwaves have been proposed. In addition, appropriate strategies have been set up to separately isolate the free and bound fractions phenolics.

Taking a look to the wide range of analytical procedures devoted to the determination of polyphenols in rice extracts, it appears clear that the most critical aspect is represented, in most cases, by the adoption of separated approaches aimed to definition of the quali-quantitative profiles of the various classes of analytes. Considerable steps forward have been made in the elucidation of the chemical structures of these bioactive compounds but whilst mass spectrometry-based techniques certainly represent a powerful tool for the definition of the brown rice phenolic profile, the absence of complete validation protocols for most of the proposed procedures prevent us to assess the actual reliability of the quantitative data provided. 

In the same way, the simple and rapid spectrophotometric procedures designed for the quantification of the total content of specific classes of polyphenols and for the evaluation of the antioxidant properties, although well established, lack any type of information regarding the precision and accuracy of the data. 

It is our strong belief that the research efforts made up to now constitute a great starting point towards the development of analytical tools aimed to investigate on the phenolic fraction of whole grain rice but, on the basis of the procedures already assessed, future research efforts should be focused on the development of methods aimed to the comprehensive determination of different classes of compounds and the assessment of reliability of the quantitative data.

## Figures and Tables

**Table 1:** Selected HPLC methods for the determination of phenolic compounds in rice.

Chromatographic Technique	Stationary Phase	Mobile Phase	Quantitative Analysis	Validation	Samples Origin	Target Analytes	Reference
HPLC-DAD	Kinetex C-18 (150 × 4.6 mm, 5 µm)	A: 0.1% Formic acid in water B: 0.1% Formic acid in acetonitrile C: 0.1% Formic acid in methanol	y	y	Rice from Brazil	Gallic acid; Protocatechuic acid; 4-Hydroxybenzoic acid; Catechin; Vanillic acid; Caffeic acid; Chlorogenic acid; Syringic acid; Epicatechin; *p*-Coumaric acid; *trans*-Ferulic acid; Sinapic acid; Kaempferol-3DGlp; Myricetin; Resveratrol, *trans*-Cinnamic acid; Quercetin; Kaempferol.	[35]
HPLC-DAD	Zorbax SB-CN (150 × 3 mm, 3.5 µm)	A: 0.2% Phosphoric acid B: Water C: Acetonitrile	y	n	Kamlaing black rice from Thailand	Caffeic acid; *p*-Coumaric acid; Ferulic acid; Gallic acid; Protocatechuic acid; Hydroxybenzoic acid; Sinapic acid; Vanillic acid; Syringic acid; Cyanidin-3-glucoside; Cyanidin (aglycone)	[41]
1. HPLC-ESI(+)-MS/MS (*qualitative*) 2. HPLC-APCI(+)-MS (*quantitative*)	Zorbax Eclipse (100 × 3 mm, 3.5 µm)	A: 0.5% Formic acid B: Methanol	y	n	Four varieties of black rice from Thailand	Cyanidin 3-glucoside; Peonidin 3-glucoside	[26]
1. HPLC-ESI(−)-MS/MS (*qualitative*) 2. HPLC-DAD (*quantitative*)	*Phenolic acids* Zorbax Eclipse XDB C18 (150 × 4.6 mm, 5 µm)	*Free phenolic acids* A: 2.5% Methanol + 0.5% Formic acid in water B: Methanol *Bound* *phenolic acids* A: 50 mM Phosphoric acid at pH 2.5 B: Acetonitrile	y	n	Non-glutinous purple rice from Thailand	Protocatechuic acid; Vanillic acid; *p*-Coumaric acid; Ferulic acid; Gallic acid; *p*-Hydroxybenzoic acid	[23]
1. HPLC-ESI(+)-MS/MS (*qualitative*) 2. HPLC-DAD (*quantitative*)	*Anthocyanins* Symmetry C18 column (75 × 4.6 mm, 3.5 µm)	*Anthocyanins* A: 5% Formic acid in water B: 5% Formic acid in acetonitrile	y	n		Cyanidin-3-glucoside; Peonidin-3-glucoside	
HPLC-DAD-APCI(+/−)-MS	*Phenolic acids* Zorbax SB-Aq (250 × 4.6 mm, 5 µm)	*Phenolic acids* A: 2.5% Formic acids in water B: Methanol	n	n	Eight red-grain and three light brown-grained rice varieties from Sri Lanka	Ferulic acid; *p*-Coumaric acid; Sinapic acid; Caffeic acid	[10]
	*Proanthocyanidins* Ascentis C18 (250 × 4.6 mm, 5 µm)	*Proanthocyanidins* A: 0.3% Formic acid in water B: 0.1% Formic acid in methanol	n	n		Dimers and trimers	
1. HPLC-DAD (*Phenolic acids*) 2. HPLC-DAD-ESI(−)-MS/MS (*Identification of unknown peaks*)	*1. Phenolic acids* Atlantis dC18 (250 × 4.6 mm, 5 µm) *2. Unknown peaks* Hydrosphere C18 HS-3C2 (150 × 2.0 mm, 5 µm)	*1. Phenolic acids* A: 0.1% Formic acid in water B: Methanol *2. Unknown peaks* *A:* 0.1% Formic acid in water B: 0.1% Formic acid in Methanol	y	n	Colored rice bran from six rice samples collected from the local markets	Protocatechuic acid; *p*-Hydroxybenzoic acid; Vanillic acid; *p*-Coumaric acid; Ferulic acid; Sinapic acid; Protocatechualdehyde.	[42]
3. HPLC-DAD-ESI-MS/MS (*Anthocyanins*)	YMC-pack ODS-AQ (250 × 4.6 mm, 5 µm)	A: 0.1% Formic acid in water B: Methanol	y	n		Cyanidin 3-glucoside; Peonidin 3-glucoside; Cyanidin 3-rutinoside	
HPLC-DAD	Kinetex C18 (150 × 4.6 mm, 2.6 µm)	A: 1% Acetic acid in water B: 1% Acetic Acid + 32% Acetonitrile in water	y	n	Four samples of Zizania aquatica L. purchased in local markets in Czech Republic	Chlorogenic acid; Gallic acid; Protocatechuic acid; *p*-Hydroxybenzoic acid; Vanillic acid; Caffeic acid; Syringic acid; *p*-Coumaric acid; Ferulic acid; Sinapic acid; Ellagic acid; *o*-Coumaric acid; Protocatechuic ethyl acid; Cinnamic acid; Epigallocatechin; Catechin; Epicatechin; Rutin; Quercetin; Kaempferol.	[14]
HPLC-VWD	Zorbax SB-C18 (250 × 4.6 mm, 5 µm)	A: 0.4% Acetic acid B: Acetonitrile	y	n	Fresh brown rice from China	Protocatechuic acid; Chlorogenic acid; Caffeic acid; Syringic acid; Coumaric acid; Ferulic acid.	[39]
HPLC-VWD	Zorbax SB-C18 (250 × 4.6 mm, 5 µm)	A: 0.4% Acetic acid B: Acetonitrile	y	n	Indica cultivar Yinfengxue and Japonica cultivar Wujingyun 27 from China	Protocatechuic acid; Chlorogenic acid; Caffeic acid; Syringic acid; Coumaric acid; Ferulic acid.	[15]
HPLC-DAD	C18 (150 × 4.6 mm, 5 µm)	A: 0.1% Trifluoracetic acid in water B: 0.1% Trifluoroacetic acid in acetonitrile	y	n	Rice from Portugal	Gallic acid; Protocatechuic acid; *p*-Hydroxybenzoic acid; Vanillic acid; Syringic acid; Chlorogenic acid; Caffeic acid; *p*-Coumaric acid; Sinapic acid; Ferulic acid. Luteolin-7-*O*-glucoside; Apigenin-7-*O*-glucoside; Apigenin; Tricin.	[16]
HPLC-DAD	LUNA C-18 (250 × 4.6 mm, 5 µm)	A: 3% Acetic acid in water B: 3% Acetic acid and 25% Acetonitrile in water	y	n	Paddy-rice samples from Thailand	4-Hydroxybenzoic acid: Gallic acid; Protocatechuic acid; *p*-Hydroxybenzoic acid; Vanillic acid; 6-Hydrocinnamic acid: Chlorogenic acid; Caffeic acid; Syringic acid; *p*-Coumaric acid; Ferulic acid; Sinapic acid. Rutin; Myricetin; Quercetin; Apigenin; Kaempferol	[21]
1. HPLC-DAD (*Phenolic acids and anthocyanins*)	Zorbax SB-C18 (150 × 4.6 mm, 3.5 µm)	*Phenolic acids* A: 0.1% Formic acid in water B: 0.1% Formic acid in Acetonitrile *Anthocyanins* A: 6% Formic acid in water B: Methanol	y	n	Six rice cultivars from Texas	Protocatechuic acid; Vanillic acid; *p*-Coumaric acid; Ferulic acid; Sinapic acid. Cyanidin 3-galactoside; Cyanidin 3-glucoside; Cyanidin 3-rutinoside; Peonidin 3-glucoside.	[36]
2. HPLC-FD (*Proanthocyanidins*)	Develosil Diol (250 × 4.6 mm, 5 μm)	A: 2% Acetic acid in acetonitrile B: 2% Acetic acid + 3% water in acetonitrile	y	n		Monomers to decamers	
1. HPLC-DAD-ESI(−)-MS/MS *(Phenolic acids)* 2. HPLC-DAD-ESI (+)-MS/MS *(Anthocyanins)*	1. RP 18 (250 × 4.6 mm, 5 µm) 2. Gemini C18 110A (150 × 4.6 mm, 5 µm)	*1. Phenolic acids* A: 0.1% Acetic acid in water B: 0.1% Acetic acid in methanol *2. Anthocyanins* A: 0.5% Formic acid in water B: 0.5% Formic acid in methanol	y	n	White, red and black rice from China	Protocatechuic acid; Vanillic acid; *p*-Hydroxybenzoic acid; Syringic acid; *trans*-*p*-Coumaric acid; *trans*-Sinapic acid; Ferulic acid. Cyanidin 3-glucoside; Peonidin 3-glucoside; Cyanidin 3-rutinoside	[19]
HPLC-DAD	Intersil ODS-3 (150 × 4.6 mm, 5 μm)	A: Trifluoroacetic acid in water (pH 2.5) B: Acetonitrile	y	n	Various rice varieties from Iran	Gallic acid; Salicylic acid; Caffeic acid; Pyrogallol; Quercetin; Rutin; Myricetin; Kaempferol; Naringin; Apigenin; Genistein; Daidzein	[18]
HPLC-DAD	RP 18 LiChroCART (250 × 4 mm, 5 µm)	A: 2% Acetic acid and 5% Methanol in water B: 2% Acetic acid and 88% Methanol in water	y	y	Commercial rice samples from Spain	Protocatechuic acid; Vanillin; Protocatechuic aldehyde; *p*-Hydroxybenzoic acid; *p*-Hydroxybenzaldehyde; Ferulic acid; Sinapic acid; Guaiacol; *p*-Coumaric Acid; Caffeic acid; 5-Hydroxymethyl-2-furaldehyde; Furfural; 5-Methylfurfural; Syringic Acid; Ellagic acid	[34]
1. HPLC-DAD-ESI(+/−)-MS/MS *(Hydroxycinnamic acids and flavonols)*	Zorbax Eclipse XDB-C18 (150 × 2.1 mm, 3.5 μm)	A: 3% Acetonitrile and 8.5% Formic acid in water B: 50% Acetonitrile and 8.5% Formic acid in water C: 90% methanol and 8.5% formic acid in water	y	n	White, red and black cultivars from Italy	3-*p*-Coumaroylquinic acid; 3-Feruloylquinic acid; 4-*p*-Coumaroylquinic acid; 4-Feruloylquinic acid; Quercetin 3-glucoside; Quercetin 3-rutinoside; Isorhamnetin 3-glucoside; Isorhamnetin 3-rutinoside; Quercetin; Isorhamnetin; Diidroquercetin 3-glucoside; Diidroisorhamnetin-3-glucoside.	[27]
2. HPLC-DAD-ESI -MS/MS (*Anthocyanins*)		A: 3% Acetonitrile and 10% Formic acid in water B: 50% Acetonitrile and 10% Formic acid in water				Cyanidin 3-glucoside; Peonidin 3-glucoside; Cyanidin 3-gentioside; Cyanidin 3-rutinoside; Malvidin 3-glucoside; Peonidin 3-rutinoside.	
3. HPLC-DAD-ESI -MS/MS (*Flavan-3-ols*)		A: 1% Formic acid and 2% Methanol in water B: Methanol				Catechin; Epicatechin; Gallocatechin; Epigallocatechin (monomers and dimers)	
HPLC-DAD-ESI(+/−)-MS	Zorbax Eclipse plus (150 × 4.6 mm, 5 µm)	A: Acetonitrile B: 2% Acetic acid in water	n	n	Black rice from China	Cyanidin 3-sophoroside; Cyanidin 3-glucoside; Peonidin 3-glucoside; Procyanidin glucoside; Caffeic acid hexose; Procyanidin B2-3-*O*-gallate hexose;Epiafzelchin-epicatechin-*O*-dimethylgallate.	[49]
